# Cost-Effectiveness and Validity Assessment of Cyscope Microscope, Quantitative Buffy Coat Microscope, and Rapid Diagnostic Kit for Malaria Diagnosis among Clinic Attendees in Ibadan, Nigeria

**DOI:** 10.1155/2016/5242498

**Published:** 2016-07-14

**Authors:** Abiodun Ogunniyi, Magbagbeola David Dairo, Hannah Dada-Adegbola, Ikeoluwapo O. Ajayi, Adebola Olayinka, Wellington A. Oyibo, Olufunmilayo I. Fawole, Olufemi Ajumobi

**Affiliations:** ^1^Nigeria Field Epidemiology and Laboratory Training Program, Abuja 900231, Nigeria; ^2^Department of Medical Microbiology, University College Hospital, Ibadan 200281, Nigeria; ^3^Department of Epidemiology and Medical Statistics, College of Medicine, University of Ibadan, Ibadan 200281, Nigeria; ^4^Department of Medical Microbiology, Ahmadu Bello University, Zaria 810105, Nigeria; ^5^Department of Medical Microbiology, College of Medicine, University of Lagos, Lagos 100283, Nigeria; ^6^National Malaria Elimination Program, Federal Ministry of Health, Abuja 900211, Nigeria

## Abstract

*Background.* Unavailability of accurate, rapid, reliable, and cost-effective malaria diagnostic instruments constitutes major a challenge to malaria elimination. We validated alternative malaria diagnostic instruments and assessed their comparative cost-effectiveness.* Method.* Using a cross-sectional study design, 502 patients with malaria symptoms at selected health facilities in Ibadan between January and April 2014 were recruited consecutively. We examined malaria parasites using Cyscope®, QBC, and CareStart*™* and results were compared to light microscopy (LM). Validity was determined by assessing sensitivity, specificity, positive predictive value (PPV), and negative predictive value (NPV). Costs per hour of use for instruments and turnaround time were determined.* Result.* Sensitivity of the instruments was 76.0% (CareStart), 95.0% (Cyscope), and 98.1% (QBC). Specificity was 96.0% (CareStart), 87.3% (Cyscope), and 85.5% (QBC). PPV were 65.2%, 67.5%, and 84.7%, while NPV were 93.6%, 98.6%, and 99.4% for CareStart, Cyscope, and QBC with Kappa values of 0.75 (CI = 0.68–0.82) for CareStart, 0.72 (CI = 0.65–0.78) for Cyscope, and 0.71 (CI = 0.64–0.77) for QBC. Average cost per hour of use was the lowest ($2.04) with the Cyscope. Turnaround time was the fastest with Cyscope (5 minutes).* Conclusion.* Cyscope fluorescent microscope had the shortest turnaround time and is the most cost-effective of all the malaria diagnostic instruments evaluated.

## 1. Background

The world malaria report of 2014 showed that, as at 2013, millions of people at risk of malaria did not have access to interventions such as insecticide treated mosquito nets (ITNs), diagnostic testing, and artemisinin-based combination therapies (ACTs). As a result, 198 million cases and 584 000 deaths occur every year [1]. In Nigeria, malaria is the major cause of morbidity and mortality, especially among children less than five years [2]. Malaria is a social and economic problem, which consumed about US$ 3.5 million in government funding and US$ 2.3 million from other stakeholders in the form of various control attempts in 2003 [3]. In Sub-Saharan Africa (SSA), where 91% of all malaria-related deaths take place, malaria is estimated to result in an annual loss of 35.4 million Disability Adjusted Life Years with 85% of the deaths among children below five years. In SSA, around 40% of all public health spending is related to malaria [3]. Malaria is responsible for about a 1.3% reduction in the average annual rate of economic growth for those countries with the highest burden [4].

Poor diagnosis hinders effective malaria control in many countries of SSA with associated economic impact. This is due to a combination of factors, including nonspecific clinical presentation of the disease, high prevalence of asymptomatic infection in some areas, lack of resources, insufficient access to trained health care providers and health facilities, and widespread practice of self-treatment for symptoms suggestive of malaria [5]. One of the main interventions of the Global Malaria Control Strategy is the prompt and accurate diagnosis of the disease which is vital for effective case management [6].

Until recently, conventional Giemsa-stained light microscopy being the mainstay of malaria parasitological diagnosis in the absence of rapid tests is a major contributing factor to presumptive diagnosis. Though a valuable technique when performed correctly, it is unreliable and wasteful when poorly executed [7]. Challenges associated with laboratory diagnosis using light microscopy include lack of skilled microscopists, variation in individual training and/or experience, limited supply of microscopes and reagents as well as variation in equipment maintenance, and inadequate quality control [8], which discourages implementation of the World Health Organisation (WHO) guideline of parasite-based diagnosis before malaria treatment.

The introduction of newer and more rapid diagnostic techniques like Cyscope fluorescent microscopy (Cyscope), quantitative buffy coat fluorescent microscopy (QBC), and CareStart (a histidine-rich protein-2-based rapid diagnostic test (RDT) kit) was meant to overcome the challenges of using light microscopy. There is a need, however, to assess the performance and cost-effectiveness of these tools in order to guide further malaria management interventions in endemic and resource limited settings like Nigeria. This study was conducted to compare the diagnostic performance characteristics and cost-effectiveness of Cyscope (Partec, Görlitz, Germany), quantitative buffy coat (QBC, Becton Dickinson, Le Pont de Claix, France), and CareStart (AccessBio, Inc., New Jersey, USA) (a recently introduced RDT into the Nigeria market) for malaria parasite detection among clinically suspected malaria cases in a malaria-endemic environment.

## 2. Methods

Malaria transmission has been intense, stable, and holoendemic in most parts of the country. Recent reports suggest some changes over time, such that, as at 2010, 85% of Nigerians lived in areas supporting mesoendemic transmission and 15% lived under conditions of hyper-holo-endemicity, with some areas within FCT and northeastern states supporting hypoendemicity [9]. Seasonality, intensity, and duration of malaria transmission in Nigeria vary according to five ecological strata. These include mangrove swamps, rain forest, guinea-savannah, Sudan-savannah, and Sahel-savannah. The duration of the season decreases from the south to the north, being perennial in duration in most of the south but lasting 3 months or less in the northeastern region bordering Chad [10]. In the north, high transmission season is between July and November and in the south, including southwest the study area, it is between April and October with bimodal peaks in July and September.

We carried out an evaluative cross-sectional study at the outpatient units of four health facilities in Ibadan, southwest Nigeria, between January 1 and April 30, 2014. The study sample size of 502 was calculated using a prevalence of 50% (due to nonavailability of published similar study as at the time of this study) with precision of 5% at 95% confidence interval and design effect of 1.5.

We selected four health facilities comprising one tertiary health facility (University College Hospital), one secondary health facility (Adeoyo State Hospital), and two primary health facilities (Kola Daisi Comprehensive Health Centre and Remi Babalola Health Centre) by simple random sampling in Ibadan southwest Nigeria as study sites. At the phlebotomy units of these facilities, patients suspected by clinicians to have malaria who presented with laboratory forms requesting malaria parasite investigation and consented to participate were recruited consecutively until the sample size of 502 was achieved. We defined a suspected case of malaria as a patient of any age for whom a clinician has made a decision to treat malaria with or without a history of fever or axillary body temperature ≥ 37.5°C.

Certified phlebotomists, following standard operating procedure, collected 1 mL of venous blood sample from each participant by venipuncture into an EDTA bottle. The specimen was tested for malaria parasites using the four diagnostic instruments, namely, light microscope, CareStart RDT, quantitative buffy coat, and Cyscope. The patients' biodata and the results of the performance of all the malaria diagnostic instruments were collected using a structured data collection* proforma* register.

### 2.1. Light Microscopy (LM)

A WHO certified malaria microscopist examined the Giemsa-stained thick and thin films with a Zeiss light microscope (Axiostar Plus, Carl Zeiss Microimaging, Germany) using the high power (40x) and the oil immersion (100x) objectives. Another WHO certified microscopist double checked the results. These microscopists, who hold bachelor degree in Medical Laboratory Science, were blinded to the outcome of other diagnostic instruments.

### 2.2. Cyscope Fluorescent Microscopy

A trained and experienced medical laboratory scientist was engaged in the use of Cyscope malaria fluorescent microscope (CY-S-4005U, Partec GmbH, Am Flugplatz 13, 02828 Görlitz, Germany). Using the manufacturer's instruction, 10 *μ*L of blood sample from each patient was applied to the preprepared slides of the fluorescent microscope, wet mounted with fluorescein dye, and viewed with the microscope. Presence (or absence) of malaria parasites was confirmed by viewing the fluorescent DNA of* plasmodia* under the microscope using ×40 objective.

### 2.3. Quantitative Buffy Coat Fluorescent Microscopy

A trained medical laboratory scientist with seven years of experience conducted QBC malaria test using QBC ParaLens Advance (QBC Diagnostics, Port Matilda, PA 16870, USA) according to the manufacturer's instruction.

### 2.4. CareStart Rapid Diagnostic Kit

A trained medical laboratory scientist also tested individual specimens with CareStart, a histidine-rich protein-2 (HRP-2) RDT (specific for* Plasmodium falciparum* species) manufactured by Access Bio, Inc., New Jersey, USA, with Lot no. MO3B10 and expiry date July 2015. Manufacturer's instruction on standard operating procedures was followed.

## 3. Turnaround Time

We calculated the turnaround time (TaT) as the time interval between receipt of the sample by the laboratory personnel who processed it and the time when results were generated following the standard operating procedure for each diagnostic instrument. The TaT of each procedure was monitored by trained research assistants using stopwatches and recorded in a structured data collection register.

### 3.1. Cost-Effectiveness Analysis

Cost-effectiveness analysis was calculated as the ratio of the resources used to the related effects, which is classified by comparison of the costs/input and consequences/outcome. The consensus paper on Guidelines on Health Economic Evaluation, Institut für Pharmaökonomische, Austria, was used for this analysis.

### 3.2. Cost/Input

The costs were separated into categories, namely, equipment, consumables cost, personnel, and miscellaneous items such as electricity.

#### 3.2.1. Equipment

We made the following assumptions in costing the equipment: (i) that machine cost is per unit time of use, assuming uniform depreciation over time/lifespan of the equipment (fixed lifespan is 3 years for the entire machine), and (ii) that period of use was fixed at 8 hours per day.

Machine cost per unit time of use = acquisition cost/total lifespan of equipment in real use days (i.e., excluding weekends during which period the facilities do not work or offer skeletal services).

#### 3.2.2. Reagents and Consumables

Cost of reagents/consumables was calculated per session of use (8 hours/day).

#### 3.2.3. Personnel

Personnel cost per unit time = standard monthly wage/hour, expressed in wage per hour. Basic staff qualification for CareStart usage is post-primary education while the staff qualification for other diagnostic tools is post-secondary education. Using the United States Office of Personnel Management, General Schedule Qualification Standards, 2014 (GS), given 8 hours/day and 21 working days, is as reflected in Table 4. Electricity was estimated at $0.08/hour (equivalent of N12.99).

### 3.3. Consequences/Output

It was expected that each diagnostic tool produces a result (yield) for the malaria parasite test as the output or consequence of the use:

(1) $$\begin{array}{c} \text{The cost per hour}=\frac{\text{total cost of input in 1 hour}}{\text{number of tests per hour}}. \end{array}$$

### 3.4. Quality Control

Standard operating procedures (SOPs) were developed and validated for venipuncture and all laboratory procedures ensuring compliance with international practicing standard. Laboratory procedures were repeated by another experienced professional for each of the tools and a tie breaker observer was engaged where there were conflicting results. Data entered was double checked by the laboratory supervisor in charge to ensure consistency.

### 3.5. Data Management and Analysis

Microsoft Excel (2008) and Epi Info version 7 were used for data entry, data cleaning, and analysis. We summarized data using proportions and means. Statistical analyses of the comparison of the diagnostic performance of the three methods of laboratory diagnosis of malaria parasites were carried out by assessing the sensitivity, specificity, and predictive values of all the instruments using light microscopy as a gold standard and inter-instrument agreement was analyzed using Kappa statistics at 5% level of significance.

## 4. Result

Malaria parasitaemia prevalence in all the 502 samples was 21.7%, 19.5%, 30.7%, and 32.7% by LM, CareStart, Cyscope, and QBC, respectively.* Plasmodium falciparum* constituted 99% of malaria parasite species detected by LM. The sensitivity of the instruments compared with LM was 76% (CareStart), 95% (Cyscope), and 98.1% (QBC), while specificity was 96% (CareStart), 87.3% (Cyscope), and 85.5% (QBC). Positive predictive value was 84.7% for CareStart, 67.5% for Cyscope, and 65.2% for QBC, and negative predictive value was 93.6% (CareStart), 98.6% (Cyscope), and 99.4% (QBC) (Table 1).

For inter-instrument agreement, all the diagnostic instruments tested, CareStart, Cyscope, and QBC, had good agreement with the light microscopy as shown by Kappa values of 0.71 (CI = 0.64–0.77), 0.72 (CI = 0.65–0.78), and 0.75 (CI = 0.68–0.82), respectively.

The mean turnaround time for LM was 45.0 (standard deviation (SD): 1.9) minutes; for CareStart it was 17.4 (SD: 1.7) minutes; for Cyscope it was 4.5 (SD: 1.0) minutes; and for QBC fluorescent microscopy it was 8.5 (SD: 1.2) minutes (Table 2). Per hour, the LM yielded one test result; CareStart yielded three, while Cyscope and QBC yielded approximately 12 and 7 test results, respectively.

The cost-effectiveness ratio of LM was $10.77 per test; for CareStart it was $16.82 per test; for Cyscope fluorescent microscopy it was $2.04 per test; and for QBC fluorescent microscopy it was $5.89 per test (Table 3).

## 5. Discussion

This study validated three malaria parasites diagnostic test tools in comparison with light microscopy. Cyscope and QBC microscopes had sensitivity of 95% and above. This finding is in concordance with earlier studies in Nigeria and Uganda on sensitivity of diagnostic tools for malaria parasite detection which showed that QBC and Cyscope demonstrated higher sensitivity and CareStart lower sensitivity when compared with light microscopy [11, 12]. However, CareStart showed better specificity and PPV than other instruments, which could tend to forestall inappropriate antimalarial treatment. Previous studies conducted using different HRP-2 rapid diagnostic kits for* P. falciparum* only in northeastern Tanzania and in Uganda however have reported sensitivities of 95.4% and 97.6% for Paracheck and Paracheck pf, respectively [13, 14]. CareStart was validated in this study as a new introduction to the country's diagnosis tools.

The absolute necessity for rational therapy in the face of ever increasing occurrence of drug resistance places increasing importance on the accuracy of malaria diagnosis [15]. Currently, Giemsa microscopy and rapid diagnostic tests (RDTs) are the two diagnostics which seem to have the largest impact on malaria control [16, 17].

Cyscope sensitivity complied with 95% WHO standard for sensitivity of rapid diagnostic test (RDT) [18]. This finding compares with studies by Hassan et al., 2011, and Nkrumah et al., 2011, from Sudan and Ghana, respectively, which demonstrated sensitivity of 97.4% and 100%, respectively, for Cyscope [19, 20]. However, Sousa-Figueiredo et al., 2010, and Rabiu et al., 2013, from Uganda and Nigeria reported lower sensitivity (92.1%  and 91.3%, resp.) [11, 12]. The latter study was done in field setting and subjected to likelihood of higher false positives.

With respect to the operational characteristics of the tools, while light microscopy will produce about one result per hour, CareStart will produce three, and QBC and Cyscope will produce seven and twelve results per hour, respectively. Light microscopy is dependent on availability of electrical power for functionality. Only the Cyscope microscope has a standby battery as an alternative source of electric current.

Light microscopy, QBC, CareStart, and Cyscope costs are $10.77, $5.89, $5.61, and $2.04, respectively, per hour of use. Considering the cost per hour of use of other tools, the cost of a test result from light microscopy is equivalent to cost of five test samples examined by Cyscope. In the same vein, costs of each of QBC and CareStart are equivalent to the cost of three test samples examined by Cyscope. Light microscopy, aside relatively cheap reagents, is the least cost-effective of all the instruments.

We found that Cyscope fluorescent microscope is a reliable diagnostic tool that is very sensitive and specific in diagnosing* falciparum* malaria, the predominant* Plasmodium* species (94.8%) in Nigeria, which was corroborated in this study. Cyscope microscope for malaria detection is currently designed to detect only* falciparum* species. However, studies by Sousa-Figueiredo et al. from Uganda found that Cyscope detected all* Plasmodium* species but could not differentiate between them. Relatively high false positives were recorded in its usage from Uganda and Sudan which were attributed to small fluorescent bodies of unknown origin from the community/field mistaken as malaria parasites and submicroscopic parasitaemia among some pregnant women [11, 19]. Further studies are recommended to assess its effectiveness in differentiating other* Plasmodium* species. Although QBC has the best sensitivity and can detect all* Plasmodium* spp., the cost-effectiveness analysis and operational characteristics showed that its applicability may be more relevant in tertiary health care facilities.

As with other diagnostic instruments, CareStart demonstrated best specificity and PPV while Cyscope showed better sensitivity and NPV. Cyscope microscope is a new technology that involves the use of lyophilized DNA probe in a fluorescein dye mounted on glass slide using wet preparation to detect malaria parasite in blood sample within 5 minutes, although it has likely chances of false positives especially in uncontrolled field setting [11]. Its performance feature of high sensitivity, being cost-effective, and its requirement of electrical supply lend credence to its suitability for programmatic deployment at health care settings.

The Engineering and Physical Sciences Research Council, United Kingdom, holds that all new points of care diagnostics, including diagnostics for infectious diseases, should meet the ASSURED criteria, that is, Affordable by those at risk of infection, Sensitive with very few false negatives, Specific with very few false positives, User-friendly tests that are simple to perform and require minimal training, Rapid, to enable treatment at first visit, and Robust, for example, not requiring refrigerated storage, Equipment-free, and Delivered to those who need it. Based on these criteria and the need to expand malaria diagnostic services as part of a greater framework of health system strengthening within resource-limited settings [21], Cyscope fits in as a point-of-care diagnostic device for health care settings in resource-limited malaria endemic areas. CareStart RDT also does fit in the criteria, but our finding demonstrated that Cyscope is better than RDT in some of the criteria.

## 6. Conclusion

The findings of this study showed that CareStart, QBC fluorescent microscopy, and Cyscope fluorescent microscopy are valuable complements to light microscopy because they help expand the coverage of parasite-based diagnosis and minimize presumptive diagnosis. The cost of improved malaria diagnosis will inevitably increase, by investment in whether light microscopy or CareStart or both. However, such investment offers a more promising cost-effective strategy with the deployment of Cyscope fluorescent microscopy in appropriate settings.

Cyscope fluorescent microscope had the least turnaround diagnostic time and the highest throughput and it is the most cost-effective of all the laboratory diagnostic instruments evaluated. We therefore strongly recommend Cyscope fluorescent microscope for malaria parasite detection especially in secondary and tertiary health care facilities where the request for malaria diagnosis is increasingly high.

## Figures and Tables

**Table 1:** Personnel's salary per hour required for the equipment tested, 2014.

GS level: Nigeria's equivalence	Equipment requirement	Salary per hour in $	Salary per hour in N
GS 2: secondary school	CareStart	12.50	2475
GS 5: university degree	Cyscope	17,10	3385.8
GS 7: professional degree	QBC Light microscope	21.14	4185.72

**Table 2:** Diagnostic accuracy of CareStart, Cyscope fluorescent microscopy, and QBC fluorescent microscopy, Ibadan, April 2014.

Diagnostic instrument	Sensitivity	Specificity	PPV	NPV
	% (95% CI)	% (95% CI)	% (95% CI)	% (95% CI)
CareStart	76 (72.3–79.7)	96 (94.3–97.7)	84.7 (81.4–87.8)	93.6 (91.5–95.7)
Cyscope	95 (93.1–96.9)	87.3 (84.4–90.2)	67.5 (63.4–71.6)	98.6 (97.6–99.6)
QBC	98.1 (96.9–99.3)	85.5 (82.4–88.6)	65.2 (61.0–69.4)	99.4 (98.7–100.1)

**Table 3:** Operational characteristics of CareStart, light, Cyscope, and QBC microscopes, Ibadan, April 2014^*∗∗*^.

Parameters	CareStart	Light microscope	Cyscope	QBC
Turnaround time	20 minutes	45 minutes	5 minutes	8 minutes
Blood quantity needed/test	3 uL	10 uL	8 uL	50 uL
Electric current by standby battery	NA^*∗*^	No	Yes	No
Average cost of equipment	NA^*∗*^	$1,197	$1,155	$14,970
Number of tests/hour	3	1	12	7

^*∗∗*^Findings as of March 2014 obtained from this study's procedures and manufacturer's website. ^*∗*^NA: not applicable.

**Table 4:** Cost-effectiveness ratio of CareStart, light microscope, Cyscope, and QBC, April 2014.

Input	CareStart	Light microscopy	Cyscope	QBC
Equipment cost ($)	NA^*∗*^	1,197	1,155	14,970
Equipment cost per unit time of use ($/hr)	NA^*∗*^	0.2	0.2	2.5
Reagent/consumables ($/test/hr)	4.32	0.12	7.2	11.4
Personnel ($/hr)	12.5	21.14	17.10	21.14
Electricity ($/hr)	NA^*∗*^	0.08	0.03	0.23

Total cost per test ($)	5.61	10.77	2.04	5.89

^*∗*^NA: not applicable.

## Additional Points

This study was carried out during the malaria low transmission season and studies have shown that low parasitaemia could affect the parasite detection rate by light microscopy [22]. Hence the low parasite detection rate of light microscopy as a gold standard in this study could have reduced the validity indices of the tool tested. However, with the engagement of WHO certified and experienced microscopists and quality control in place during slide reading as mentioned earlier, we observed that, having compared with findings from other studies, this effect was insignificant. Also, we conducted this study amongst patients suspected to have uncomplicated malaria. We excluded patients who had severe malaria as they were expected to have been confirmed as having malaria and admitted to the hospital ward. A study to compare the cost-effectiveness of the diagnostic tools in clinical setting of severe malaria could be a subject of future research.
